# Quantitative Analysis and Discrimination of Partially Fermented Teas from Different Origins Using Visible/Near-Infrared Spectroscopy Coupled with Chemometrics

**DOI:** 10.3390/s20195451

**Published:** 2020-09-23

**Authors:** Tsung-Hsin Wu, I-Chun Tung, Han-Chun Hsu, Chih-Chun Kuo, Jenn-How Chang, Suming Chen, Chao-Yin Tsai, Yung-Kun Chuang

**Affiliations:** 1 Master Program in Food Safety, Taipei Medical University, Taipei 11031, Taiwan; ma47105005@tmu.edu.tw (T.-H.W.); ma47105004@tmu.edu.tw (I.-C.T.); 2 Department of Biomechatronics Engineering, National Taiwan University, Taipei 10617, Taiwan; r04631005@ntu.edu.tw (H.-C.H.); schen@ntu.edu.tw (S.C.); chaoyin@ntu.edu.tw (C.-Y.T.); 3 Tea Research and Extension Station, Council of Agriculture, Executive Yuan, Taoyuan 32654, Taiwan; kcc0204@ttes.gov.tw (C.-C.K.); tres703@ttes.gov.tw (J.-H.C.); 4 Taiwan Agricultural Mechanization Research and Development Center, Taipei 11051, Taiwan; 5 School of Food Safety, Taipei Medical University, Taipei 11031, Taiwan; 6 Nutrition Research Center, Taipei Medical University Hospital, Taipei 11031, Taiwan

**Keywords:** partially fermented tea, origin, production area, near-infrared spectroscopy, support vector machine

## Abstract

Partially fermented tea such as oolong tea is a popular drink worldwide. Preventing fraud in partially fermented tea has become imperative to protect producers and consumers from possible economic losses. Visible/near-infrared (VIS/NIR) spectroscopy integrated with stepwise multiple linear regression (SMLR) and support vector machine (SVM) methods were used for origin discrimination of partially fermented tea from Vietnam, China, and different production areas in Taiwan using the full visible NIR wavelength range (400–2498 nm). The SMLR and SVM models achieved satisfactory results. Models using data from chemical constituents’ specific wavelength ranges exhibited a high correlation with the spectra of teas, and the SMLR analyses improved discrimination of the types and origins when performing SVM analyses. The SVM models’ identification accuracies regarding different production areas in Taiwan were effectively enhanced using a combination of the data within specific wavelength ranges of several constituents. The accuracy rates were 100% for the discrimination of types, origins, and production areas of tea in the calibration and prediction sets using the optimal SVM models integrated with the specific wavelength ranges of the constituents in tea. NIR could be an effective tool for rapid, nondestructive, and accurate inspection of types, origins, and production areas of teas.

## 1. Introduction

Tea (*Camellia sinensis*) is one of the most popular drinks globally. Tea is rich in polyphenols, catechins, caffeine, theanine, and numerous other types of secondary metabolites. The antioxidant effect, blood pressure-reducing effect, cholesterol-reducing effect, and several nutritive values of tea have been reported [1]. Tea can be divided according to the degree of fermentation into unfermented tea (green tea), partially fermented tea (e.g., oolong tea), and fully fermented tea (black tea). The chemical constituents of tea vary depending on the type, and the differences may affect the taste and functionality of tea products. For instance, the calming effect of tea drinks is principally caused by theanine and other free amino acids (FAAs), and the astringency of tea drinks is principally caused by catechins, such as epicatechin (EC), epigallocatechin (EGC), epicatechin gallate (ECG), epigallocatechin gallate (EGCG), and other polyphenols.

In Taiwan, the warm and humid climate and geographical environment are suitable for the cultivation of tea trees. Over the years, partially fermented teas, such as paochong tea, oolong tea, and high-mountain tea, have been developed using complicated manufacturing processes. These teas also have high economic value. Therefore, partially fermented tea has become an essential agricultural product in Taiwan. In recent ten years, a growing number of people worldwide consume tea drinks, and the demand for Taiwanese tea has increased yearly. However, the production of Taiwanese tea is insufficient to meet demand, and most green tea, partially fermented tea, and black tea are imported. The largest proportion of imported tea comes from Vietnam, but it is also imported from other countries, including Japan, India, and China. Some imported teas are low quality or contain excessive pesticide residues. In recent ten years, an increasing number of partially fermented teas from Vietnam or China are also produced by highly similar tea processing techniques to Taiwanese teas. However, foreign teas are sometimes sold as Taiwanese tea (e.g., “fake” Taiwanese oolong tea from Vietnam or China) at high prices, despite their lack of authentic origin. It is difficult to distinguish between “real” and “fake” Taiwanese oolong teas according to the appearance, color, and texture of tea leaves even assessed by experts. This practice damages the benefits and rights of consumers and the superior brand image of Taiwanese tea. Therefore, discrimination of tea origin is essential to ensure the quality and safety of Taiwanese tea. There is a strong need to develop a rapid detection method for non-destructive discrimination of commercial partially fermented tea products from Taiwan, Vietnam, and China.

The origin of tea is usually assessed by experts based on tea appearance, infusion color, aroma, and taste, but objective and systematic scientific criteria for identification are lacking. To determine indices for identifying tea origin, the composition of chemical constituents and trace elements in tea has been investigated using high-performance liquid chromatography (HPLC) equipped with high-performance capillary electrophoresis [2], inductively coupled plasma atomic emission spectrometry (ICP-AES) [3], inductively coupled plasma mass spectrometry (ICP-MS) [4,5,6], liquid chromatograph-mass spectrometry (LC-MS) [7,8,9], micellar electrokinetic chromatography [10,11], and gas chromatography-mass spectrometry (GC-MS) [12]. However, these chemical detection methods require a high degree of technical skills, numerous reagents, considerable manpower, and time-consuming derivatization steps. Therefore, a rapid, efficient, repeatable, objective, and economical nondestructive inspection method is needed for origin discrimination of tea. For example, gas-phase infrared spectroscopy, one of the artificial olfaction sensing systems, has the potential to be helpful as a screening method for the quality control of tea because of its increased sensitivity, resolution, high signal-to-noise ratio, multiple-component analysis, and rapid measurement capabilities [13,14].

Near-infrared spectroscopy (NIR) is also a rapid nondestructive screening technique used to detect the composition and concentrations of chemical constituents in objects without destroying the sample. NIR is suitable for solid, powder, and liquid samples and can be used to repeatedly analyze a sample. Therefore, NIR has been widely applied in the inspection and analysis of food and agricultural products [15,16]. While application of NIR for origin discrimination of tea leaves appears promising, available literature still focuses mainly on green teas [17,18,19,20], black teas [21,22], or the partially fermented teas in a single country/region [23,24,25]. To date, the origin discrimination of Taiwanese and imported partially fermented teas have not been investigated using NIR. Because partially fermented tea is the principal type of tea in Taiwan, the objective of this study was to develop a method for discriminating tea origin using visible/NIR spectroscopy integrated with chemometrics and qualitative analyses. First, the three different types of tea (green tea, partially fermented tea, and black tea) were classified using NIR. Second, the origins of Taiwanese and imported teas were identified according to the results of qualitative analysis of tea leaves and powders. This is significant because preventing fraud in partially fermented tea has become imperative to protect producers and consumers from possible economic losses.

## 2. Materials and Methods

### 2.1. Tea Materials and Sample Preparation

In the present study, 69 Taiwanese and 33 imported tea samples were collected from the Tea Research and Extension Station, Council of Agriculture, Executive Yuan of Taiwan. The samples comprised nine green teas (six Taiwanese and three imported tea), 72 partially fermented teas (48 Taiwanese and 24 imported teas), and 21 black teas (15 Taiwanese and six imported teas). The Taiwanese tea samples were cultivated in northern, southern, and eastern Taiwan, and the imported teas were cultivated in Vietnam and China. The origins of the 102 samples are summarized in Table 1, where green tea, partially fermented tea, and black tea are expressed as G, P, and B, respectively. All the tea samples were packaged in aluminum foil vacuum seal bags, respectively, and then stored in a dry box before experiments. After VIS/NIR measurement, the tea samples were ground to a powder using a grinder (RT-02A; Sun-Great Technology Co., New Taipei City, Taiwan) and screened using a 100-mesh standard sieve to collect tea powder for VIS/NIR acquisition.

### 2.2. VIS/NIR Measurement and Chemical Analysis

A FOSS NIRS 6500 spectrometer (FOSS NIRSystems, Laurel, MD, USA) equipped with a small ring cup (i.d., 35 mm; depth, 8 mm) was used to measure the visible and NIR reflectance spectra of tea leaves and powders at 400–2498 nm wavelengths with 2 nm intervals. Absorbance values of the spectra were measured in a unit of log (1/*R*), where *R* is the reflectance. The spectrum of a sample was constructed using the average of 32 scans. VIS/NIR measurements of the tea samples were performed in our laboratory under constant temperature and relative humidity (RH) environmental conditions (25 ± 1 °C, 65 ± 2% RH) for stability and experimental reproducibility.

The constituents of the samples, including moisture concentration, pH value, total polyphenol (TP) concentration, FAA concentration, gallic acid (GA) concentration, and concentrations of individual catechins, were measured following the standard methods provided by the Tea Research and Extension Station, Council of Agriculture, Executive Yuan of Taiwan [26,27]. The measured values (actual values) were used as references for the qualitative and quantitative spectral analyses.

The reagents for measuring TP in tea were ferrous sulfate, potassium phosphate monobasic, potassium sodium tartrate tetrahydrate, and sodium phosphate dibasic dodecahydrate, which were purchased from Sigma-Aldrich (Sigma-Aldrich Corp., St. Louis, MO, USA). The reagents for measuring FAA concentration in tea were L-theanine (Tokyo Chemical Industry Co., Ltd., Portland, OR, USA), acetic acid (AvantorTM Performance Materials, Radnor, PA, USA), ninhydrin, tin(II) chloride (Alfa Aesar, Heysham, Lancashire, United Kingdom), sodium acetate trihydrate, and polyvinylpolypyrrolidone (PVPP; Sigma-Aldrich Corp., St. Louis, MO, USA). The standards for measuring GA, individual catechins, and caffeine were GA, (-)-gallocatechin (GC), (-)-EGC, (-)-catechin (C), (-)-EC, (-)-EGCG, (-)-gallocatechin gallate (GCG), (-)-ECG, (-)-catechin gallate (CG), and caffeine. These standards were purchased from Sigma-Aldrich (Sigma-Aldrich Corp., St. Louis, MO, USA).

After collecting the VIS/NIR spectral data of tea leaf and powder samples, 0.5 g of tea powder was measured out using a two-digit precision balance and placed in a 105 °C baking oven inside an aluminum box for 24 h. The dried tea powder was then weighed to assess the moisture concentration, and the average of three measurements was calculated for each sample. Furthermore, the tea powder underwent pretreatment for the measurements of pH, TP concentration, concentrations of individual catechins, and FAA concentration. Tea powder (0.5 g) was dissolved in 45 mL of deionized water at 90 °C and then processed in a 90 °C water bath for 20 min. After cooling the solution to room temperature using a cold bath, suction filtration was conducted to collect the clarified liquid from the solution. Then, 50 mL of clarified liquid was measured using a volumetric flask for the tea extraction solution. A pH meter (PC-420D; Corning, NY, USA) was used to measure the pH value of tea extraction solutions, and the average of three measurements was calculated for each sample.

First, TP content was measured. GA was used to prepare the standards of various concentrations and construct the calibration curves. Then, 500 μL of the tea extraction liquid was shaken and mixed into 500 μL of double-distilled water, 1 mL of ferrous tartrate solution, and 3 mL of phosphate buffer. Then, 200 μL of the solution was placed into a 96-well plate and stored away from light for 30 min, after which the optical density at the 540 nm wavelength was measured using a VersaMax Microplate Spectrophotometer (Molecular Devices, San Jose, CA, USA). The TP concentration was calculated on the basis of the calibration curves of GA standards, and the average of six measurements was calculated for each sample. Second, FAA concentration was measured through the preparation of standards of various concentrations and the establishment of the calibration curves with theanine. Then, 15 μL of tea extraction liquid was shaken and mixed in 0.15 g of PVPP for 30 min. Qualitative Filter Paper No. 1 (Advantec, Suite A Dublin, CA, USA) was then used to filter the solution and collect the clarified liquid. The 200 μL of clarified liquid was mixed evenly with 800 μL of double-distilled water, 0.5 mL of SnCl2, and 0.5 mL of ninhydrin and processed in a 100 °C water bath for 15 min. After cooling to room temperature in a cold-water bath, the solution was mixed evenly in 10 mL of 50% alcohol. The optical density at the 570 nm wavelength was measured using a VersaMax Microplate Spectrophotometer (Molecular Devices, San Jose, CA, USA), and the FAA concentration was converted using the calibration curves of theanine standards. The average of six measurements was calculated for each sample. Third, GA, individual catechins, and caffeine were measured. The tea extraction liquid filtered through a 0.45-μm filter film was measured using HPLC, a SpectraSystem P4000 pump, and a UV6000LP detector under the following conditions: the mobile phase of acetonitrile, 0.1% formic acid (FA), 20-µl injection volume, 1 mL/min flow velocity, 45 °C, and 280 nm wavelength. The average of three measurements was calculated for each sample.

### 2.3. Data Analysis

After the VIS/NIR spectra and constituent concentrations of the samples were collected, the correlations between the spectra and constituents were assessed using chemometric methods, and the spectral calibration models of each constituent were constructed. The specific wavelengths, according to the constituents with high correlations, were integrated with the qualitative analyses to discriminate the types and origins of the green, partially fermented, and black teas from Taiwan, Vietnam, and China. The qualitative analysis results of tea leaves and powders were also compared.

In the present study, a stepwise multiple linear regression (SMLR) was performed using WinISI II software (Infrasoft International, State College, PA, USA) to construct the spectral calibration models of the constituents. To build calibration models over numerous wavelengths, the SMLR algorithm chooses the most important specific wavelength from the major molecular bonding region of the objects, and the second most important specific wavelength is usually chosen from the region located in the combination of related molecular bonding or the overtone of complementary bonding and by analogy.

Because the evenness of tea powder was more favorable than tea leaf, the quantitative analyses used tea powders only. To remove the light scattering, baseline shifts, and other physical interferences on the spectra of tea powders, spectral pretreatments, such as scattering correction, smoothing, and the first derivative, were applied on the spectra of all samples before SMLR. The steps of the quantitative analysis are summarized as follows: (1) all of the samples were sorted based on the concentration of the constituent to be analyzed, (2) spectral pretreatments were performed, (3) the optimal calibration models of the constituents were constructed, and the specific wavelengths of each constituent were identified. The optimal pretreatment parameters were applied and the calibration models were performed in steps 2 and 3 with leave-one-out cross-validation (LOOCV). The prediction ability of the calibration models was evaluated according to the statistical indices, including the coefficient of determination for CV (1-VR, where VR = variance ratio) and the standard error of CV (SECV).

After finishing quantitative analyses of the tea powders, the wavelength ranges around the specific wavelengths of the constituents with high correlations with the spectra were applied to discriminate the types and origins of the green teas, partially fermented teas, and black teas from Taiwan, Vietnam, and China using the support vector machine (SVM) method. The analysis cases included (1) the identification of green tea, partially fermented tea, and black tea; (2) the identification of Taiwanese and imported partially fermented tea; (3) the identification of Taiwanese and Vietnamese partially fermented tea; (4) the identification of Taiwanese and Chinese partially fermented tea; and (5) the identification of the production areas (northern, southern, and eastern Taiwan) of Taiwanese partially fermented tea. To examine the discriminant abilities of the SVM models constructed using only the specific wavelength ranges of the tea constituents in each case, the qualitative analysis results of the SVM models built using the full-wavelength spectrum (400–2498 nm) were compared.

SVM is a type of supervised-learning classification algorithm used in the field of machine learning. The principle is to identify a hyperplane from the high-dimensional data to separate two different clusters, and larger distances between the hyperplane and the margin of each cluster are more favorable to clearly identify the samples to which each cluster belongs [28]. In this study, the radial basis kernel function (RBF) was selected according to the least error and the prior knowledge by experts. RBF kernel function parameters, namely penalty coefficient (c) and gamma (γ), were optimized by a grid-search procedure. The parameter c was applied to minimize the fitting error and the complexity of the model, while parameter γ was employed to characterize the nonlinear mapping from the input space to a high-dimensional feature space [20]. The SVM analyses of tea leaves and powders were performed using the MATLAB programs (MATLAB R2010a; The MathWorks, Inc., Natick, MA, USA) developed by our research group. The qualitative analysis was described as three steps. First, all of the samples were divided into the calibration set and prediction set at a ratio of 2:1. Both of the two sets in case 1 included green teas, partially fermented teas, and black teas. Each set in cases 2, 3, and 4 included Taiwanese and imported partially fermented teas. Both of the two sets in case 5 included the partially fermented teas from different production areas in Taiwan. Second, spectral pretreatments were processed. Third, the SVM models were built, and the identification accuracies of the calibration set and prediction set in each case were evaluated.

## 3. Results and Discussion

### 3.1. Distributions of The Constituents in Tea

The statistics regarding the constituent concentrations in the green teas, partially fermented teas, and black teas are summarized in Table 2. The between-group variances of the three types of tea were analyzed using one-way ANOVA with Tukey’s range test. The results indicated that the average moisture concentration of the partially fermented teas was 5.13%, but no significant differences were identified between the three types of tea. The pH value of tea decreased as the degree of fermentation increased. The average pH values of the green teas and partially fermented teas were approximately the same and significantly higher than the pH value of the black teas. Green teas had the highest average TP concentration, and black teas had the lowest. These observations accord with previous studies [29,30]. Notably, the standard deviation of the TP concentration in the black teas was higher than that for the other two types of tea, which indicates that the TP concentrations of various fully fermented tea may differ because of tea cultivars, climate, geographical environment, or tea processing [31]. The average FAA concentrations of the three different types of tea were similar, indicating that FAA concentrations were not affected by the differences in the three types of tea. The average GA concentration of the black teas was significantly higher than that of the other two types of tea, which accorded with prior literature [32,33,34]. Moreover, the concentrations of individual catechins exhibited similar trends as the TP concentration for the three different types of tea. The average concentrations of the seven different catechins arranged in descending order were GC, EGC, EGCG, ECG, EC, GCG, and C, which was approximately consistent with previous research [33,34,35]. The data regarding CG were not collected because of insufficient concentrations in the tea samples. Furthermore, no significant differences were identified in the average caffeine concentrations of the three different types of tea, even though that of the black teas was 55.43 mg/g [29,32,35,36,37]. Many factors such as plant variety (geographical difference), leaf age, leaf quality, and extractability may have caused this result since the origins of all tea samples were different [29,38]. The results displayed in Table 2 indicate that variations in the concentrations of TPs and individual catechins could be applied to identify green tea, partially fermented tea, and black tea [30].

The statistics concerning the constituent concentrations in the partially fermented teas from Taiwan, Vietnam, and China are displayed in Table 3; the between-group variances were analyzed using one-way ANOVA with Tukey’s range test. The average moisture concentrations in Taiwanese and Vietnamese teas were similar and significantly higher than in Chinese tea. The average pH values of Taiwanese, Vietnamese, and Chinese tea were similar, indicating that the pH value of partially fermented tea seems not to be affected by the origin of tea. There were no differences in the average TP concentration of the teas from three different origins. The results indicated that the degrees of fermentation of the teas from Taiwan, Vietnam, and China were similar because the TP concentration in tea is related to the degree of fermentation. Therefore, the concentrations of individual catechins were further analyzed to identify the differences in the teas from the three different origins. Notably, the average FAA concentration of Taiwanese teas was significantly higher than that in the teas imported from Vietnam and China, suggesting that the FAA concentration could be used to discriminate Taiwanese and imported teas. The three types of tea can be arranged in descending order as Taiwanese, Vietnamese, and Chinese tea according to their average GA concentrations. However, high standard deviations were observed in both the Taiwanese and Vietnamese teas; therefore, no significant differences were identified. The average concentrations of the seven individual catechins in Taiwanese teas were similar to those in Vietnamese teas, which were significantly higher than those in Chinese teas. The average caffeine concentrations of Taiwanese and Vietnamese teas were similar and were significantly higher than in Chinese tea. In summary, the results displayed in Table 3 indicate that the moisture concentration, pH value, TP concentration, GA concentration, concentrations of the seven individual catechins, and caffeine concentration were similar in Taiwanese and Vietnamese teas, but the FAA concentrations were significantly different. Therefore, the FAA concentration could be an index to discriminate partially fermented tea from Taiwan and Vietnam. Moreover, the moisture concentration, FAA concentration, concentrations of seven individual catechins, and caffeine concentration were significantly different between Taiwanese teas and Chinese tea, and could thus be indices to discriminate the partially fermented tea from Taiwan and China.

The statistics of the constituent concentrations in the partially fermented teas from different production areas in Taiwan (northern, southern, and eastern Taiwan) are presented in Table 3, and the between-group variances were analyzed using one-way ANOVAs with Tukey’s range tests. The northern tea had the highest moisture concentration, followed by eastern, and southern teas, but no significant differences were identified among the teas from the three different production areas. The average pH values of the three types of tea were similar, which indicated that the variation of the pH value of partially fermented tea was not affected by the production area in Taiwan. The average TP concentration of the northern teas was similar to that of the southern teas and significantly higher than that of the eastern teas because most of the collected eastern teas were red oolong tea, which is processed with a higher degree of fermentation. These findings accorded with the relationship between the TP concentration and the degree of fermentation [30]. Therefore, the partially fermented teas from eastern Taiwan can be distinguished by the TP concentration in tea. The average FAA concentration in the northern teas was similar to that in the southern teas and significantly higher than that in the eastern teas. The results indicated that the FAA concentration could be used to discriminate the teas from eastern Taiwan. The average GA concentration in the southern teas was similar to that in the eastern teas and significantly higher than that in the northern teas. However, high standard deviations were observed in both the southern and eastern teas; thus, no significant differences were identified between the teas from three different production areas in Taiwan. The northern tea had the highest average concentrations of individual catechins, followed by southern and then eastern teas. The concentrations of EGC and EC in the northern teas were significantly higher than in the southern and eastern teas because most of the samples from northern Taiwan exhibited low degrees of fermentation; concentrations in the southern teas were next highest (but not high). Therefore, the concentrations of individual catechins can be employed to discriminate the teas from northern Taiwan. The average caffeine concentrations of the northern, southern, and eastern teas were similar, indicating that the variation of the caffeine concentration in partially fermented tea was not affected by the production area in Taiwan. The partially fermented teas from northern, southern, and eastern Taiwan may not be distinguishable based on differences in the concentration of a single constituent, even though the degrees of fermentation of the three types of tea differed. Therefore, using the spectroscopic information of two or more constituents, including TP, seven individual catechins, and FAA, is essential to enhance the discriminant abilities of the SVM models in performing qualitative analyses. The aforementioned results revealed that many factors associated with different origins (e.g., climate, geographical environment, tea cultivars, and tea processing) would affect the chemical compositions of tea leaves [25,31].

### 3.2. Quantifications of The Constituents in Tea Using SMLR

**Figure 1:**
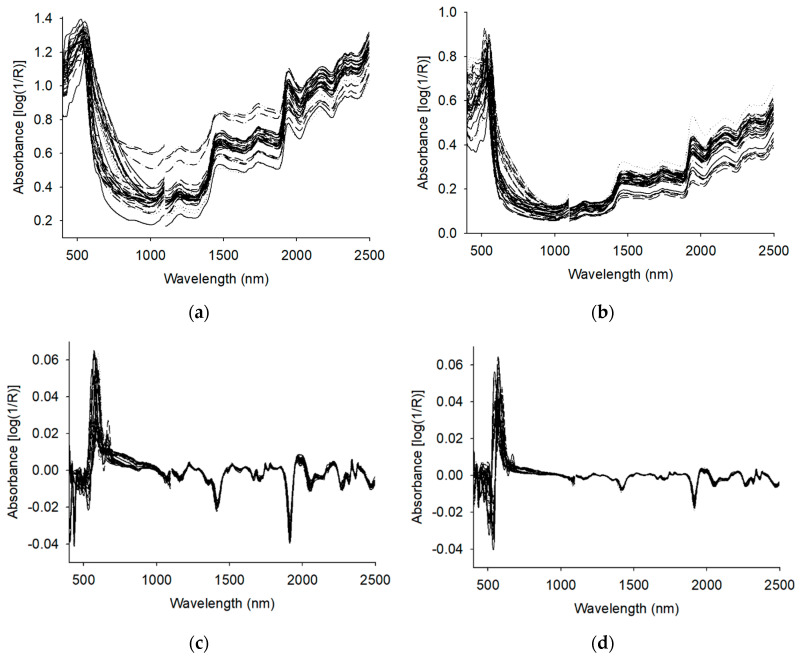
The visible/near-infrared full-wavelength spectra: (**a**) raw spectra of tea leaves, (**b**) raw spectra of tea powders, (**c**) first derivative spectra of tea leaves, and (**d**) first derivative spectra of tea powders.

The raw VIS/NIR spectra of tea leaves and powders are displayed in Figure 1a,b. The differences among the concentrations of chemical constituents in all the tea samples, including the green teas, partially fermented teas, and black teas from Taiwan, Vietnam, and China, are reflected in the variations of the absorbance and waveform in the spectra. The first derivative spectra of tea leaves and powders are displayed in Figure 1c,d, and the spectra of all samples were approximately the same. The variations in absorbance observed from the absorption peaks and troughs in the spectra were related to the concentrations of chemical constituents, including polyphenols, phenolic acids, alkaloids, and FAAs. The positions at approximately 1428 and 1940 nm are the absorption bands of O-H bonds [17,39,40], and those around 1386, 1724, 1741, 1868, and 2141 nm are the absorption bands of C-H bonds [17,40,41].

In the present study, the spectra and constituent values of the tea powders were applied to perform SMLR analyses. The spectral calibration models of the constituents were constructed, and the specific wavelengths of the constituents with high correlation were recorded. The SMLR analysis results of the constituents are listed in Table 4. The optimal calibration models for the concentrations of individual catechins were consistent with those of TP concentration. Most of the calibration models of these catechins exhibited satisfactory results, but the GCG data were not correlated with the spectra because of approximate concentrations within all of the 102 samples. The optimal calibration model of caffeine concentration revealed a 1-VR of 0.431 and SECV of 11.114 mg/g using 3 specific wavelengths (1700, 2332, and 1468 nm). Significantly lower correlation of this calibration model was caused by the caffeine concentrations in the different types of tea not being significantly different. Studies have also indicated that the relationship between caffeine concentration and the degree of fermentation of tea is nonsignificant because caffeine concentrations in different types of tea may be caused by many factors such as plant variety (geographical difference), leaf age, and leaf quality [29,38]. Our analysis results accorded with the specific wavelengths of tea constituents reported in previous research with respect to moisture concentration [21], pH value [42], TP concentration [43,44,45,46], FAA concentration [44,45], concentrations of individual catechins [18,44,45,47,48,49,50], and caffeine concentration [44,45,47,48]. Water presented absorption bands in NIR region around 970, 1450, and 1940 nm [21]. The absorption bands in the wavelength ranges from 1629 to 1792 nm and from 2049 to 2475 nm were found to be suitable for estimating TPs [44,45]. Absorption bands near 1538, 1688, and 2262 nm were correlated with FAAs [44,45]. Absorption bands around 1754, 2326–2381, 2404, and 2475 nm were found to be suitable for estimating GC [44,45,49]. Absorption bands close to 1692, 1896, 2142, 2270, and 2310 nm were correlated with EGCG [47,48]. Absorption bands near 1210, 1642, 1910, 2060, and 2475 nm were correlated with ECG [45,47,48]. Those close to 1792, 2049–2306, and 2326–2381 nm were correlated with EGC [44,45,49]; however, those near 1295, 1435–1477, 1906, 2178, 2256, 2324, 2378, and 2475 nm were correlated with EC [45,47,48,50]. The absorption bands close to 1470, 1640–1700, 1908, and 2344 nm were found to be suitable for estimating caffeine [44,45,47,48]. The results displayed in Table 4 illustrate that the specific wavelength ranges of moisture concentration, TP concentration, concentrations of individual catechins, and FAA concentration, which were highly correlated with the spectra of tea powders, could be used to discriminate the types and origins of Taiwanese and imported teas.

### 3.3. Quantitative Analyses of Tea Using SVM

The SVM analysis results of tea leaves and powders are displayed in Table 5. Case 1 investigated the different types of tea. All the samples were divided into the calibration and prediction sets at a ratio of 2:1, and thus 69 samples were categorized into the calibration set (six green teas, 48 partially fermented teas, and 15 black teas), and 33 samples were categorized into the prediction set (three green teas, 24 partially fermented teas, and six black teas). The SVM analyses of the tea leaves and powders were performed using the first derivative spectra after inverse multiplicative scatter correction (IMSC) scattering correction, with a 3-point smoothing and a gap size of 3 in derivation. The specific wavelength ranges of TP and individual catechins (450–900 nm, 1000–1400 nm, 1600–1850 nm, and 2100–2400 nm) were also used to construct the SVM models. Suitable outcomes were obtained for both models, with the identification accuracy for both the calibration and prediction sets reaching 100%. The results indicated that the green teas, partially fermented teas, and black teas could be classified effectively according to the concentration variations of TP and individual catechins instead of information on full-wavelength spectra (400–2498 nm).

Case 2 investigated the discrimination of the Taiwanese and imported partially fermented teas. The samples of partially fermented tea were divided into calibration and prediction sets at a ratio of 2:1. Thus, 48 samples were included in the calibration set (33 Taiwanese teas, 12 Vietnamese teas, and three Chinese teas), and 24 samples were categorized into the prediction set (15 Taiwanese teas, six Vietnamese teas, and three Chinese teas). The SVM analyses of the tea leaves and powders were performed using the first derivative spectra after IMSC scattering correction, with a 2-point smoothing and a gap size of 2 in derivation. Furthermore, the specific wavelength ranges of the FAA (1500–1900 nm and 2200–2300 nm) were used to construct the SVM models. Satisfactory results were obtained, with the identification accuracy for both the calibration and prediction sets reaching 100%, indicating that the Taiwanese and imported partially fermented teas could be effectively discriminated using the concentration variations of FAA. Case 3 aimed to identify Taiwanese and Vietnamese partially fermented teas. The samples of Taiwanese and Vietnamese tea were divided into calibration and prediction sets at a ratio of 2:1; thus, 45 samples were included in the calibration set (33 Taiwanese teas and 12 Vietnamese teas), and 21 samples were categorized into the prediction set (15 Taiwanese teas and six Vietnamese teas). The SVM analyses of the tea leaves and powders were performed using the first derivative spectra after IMSC scattering correction, with a 2-point smoothing and a gap size of 2 in derivation. The SVM models, constructed using full-wavelength spectra (400–2498 nm), reached 100% accuracy in identifying the calibration sets and 86% accuracy for the prediction sets because the Vietnamese tea samples with ID P24 were misclassified as Taiwanese tea. The SVM models constructed using the specific wavelength ranges of FAA (1500–1900 nm and 2200–2300 nm) yielded 100% accurate identification for both of the sets. These results indicated that the concentration variations of the FAA can be used to discriminate Taiwanese and Vietnamese partially fermented tea effectively. Case 4 aimed to identify the Taiwanese and Chinese partially fermented teas. The samples of Taiwanese and Chinese partially fermented teas were divided into calibration and prediction sets at a ratio of 2:1; thus, 36 samples were included in the calibration set (33 Taiwanese teas and three Chinese tea), and the prediction set comprised 18 samples (15 Taiwanese teas and three Chinese tea). The SVM analyses of the tea leaves and powders were performed using the first derivative spectra after IMSC scattering correction, with a 2-point smoothing and a gap size of 2 in derivation. The SVM models constructed using full-wavelength spectra (400–2498 nm) yielded 100% accurate identification for the calibration sets and 83% accuracy for the prediction sets because the Chinese tea samples with ID P17 were misclassified as Taiwanese tea, whereas the SVM models constructed using the specific wavelength ranges of individual catechins (450–900 nm, 1600–1850 nm, and 2100–2400 nm) displayed 100% accuracy in identification for both of the sets. These results suggest that the Taiwanese and Chinese partially fermented teas can be effectively discriminated using the concentration variations of individual catechins.

Case 5 focused on the discrimination of the partially fermented teas from different production areas in Taiwan. The samples of tea from northern, southern, and eastern Taiwan were divided into calibration and prediction set at a ratio of 2:1. That is, 30 samples were categorized into the calibration set (15 samples from southern Taiwan, nine samples from northern Taiwan, and six samples from eastern Taiwan), and 18 samples were included in the prediction set (nine samples from southern Taiwan, six samples from northern Taiwan, and three sample from eastern Taiwan). The SVM analyses of the tea leaves and powders were performed using the first derivative spectra after IMSC scattering correction, with a 3-point smoothing and a gap size of 3 in derivation. The SVM models constructed using full-wavelength spectra (400–2498 nm) displayed 100% accuracy in identification for the calibration sets and 83% accuracy for the prediction sets because the southern Taiwanese tea samples with ID P14 were misclassified as eastern Taiwanese tea. The SVM models constructed using the specific wavelength ranges of TP and individual catechins (450–900 nm, 1000–1400 nm, 1600–1850 nm, and 2100–2400 nm) yielded 100% accurate identification for the calibration sets and 83% accuracy for the prediction sets because the southern Taiwanese tea samples with ID P14 were misclassified as eastern Taiwanese tea. The SVM models constructed using the specific wavelength ranges of FAA (1500–1900 nm and 2200–2300 nm) yielded 100% accurate identification for the calibration sets but only 67% accuracy for the prediction sets because the southern Taiwanese tea samples with ID P14 were misclassified as eastern Taiwanese tea and the eastern Taiwanese tea samples with ID P5 were misclassified as southern Taiwanese tea. The samples of P5 and P14 were respectively misclassified as southern and eastern Taiwanese tea despite the SVM models being constructed by merging the specific wavelength ranges of TP, individual catechins, and FAA. The P5 sample was reassigned as a southern Taiwanese tea, and the sample of P14 was reassigned as an eastern Taiwanese tea, after which the SVM analyses of the tea leaves and powders were performed again. The SVM models constructed using full-wavelength spectra (400–2498 nm) yielded 100% accurate identification for the calibration sets and 83% accuracy for the prediction sets, whereas the SVM models constructed using the specific wavelength ranges of TP, individual catechins, and FAA reached 100% accuracy in identification for both sets. The results revealed that the NIR spectral characteristics of the samples of P5 and P14 were approximately the same as southern and eastern Taiwanese tea, respectively. The specific wavelength ranges of TP, individual catechins, and FAA could represent part of the information for discriminating the partially fermented teas from different production areas in Taiwan, respectively. The identification accuracies of the SVM models were enhanced by combining the specific wavelength ranges of these aforementioned constituents.

In summary, the SVM analysis results of the tea leaves and powders demonstrated that the use of spectroscopic information from the specific wavelength ranges of the chemical constituents that was highly correlated with the spectra of the tea powders contributed to success in discriminating the types and origins of Taiwanese and imported teas. Identification accuracies of the SVM models for the partially fermented teas from different production areas in Taiwan can be effectively enhanced by combining the spectroscopic data of several chemical constituents. Furthermore, the identification results from Case 5 indicate that the samples P5 and P14 could be miscategorized as southern and eastern Taiwanese tea, respectively. Disparities between the origin labeling information and the actual origin of tea may occur; however, these findings demonstrate the importance of the discriminating teas with different origins. Compared to the previous literatures with NIR spectroscopy in tea analysis [17,18,19,20,21,22,23,24,25,39,40,51], this study is the first to trace partially fermented tea samples from different countries and regions simultaneously. In terms of future research, the partially fermented tea samples from more countries or regions may be included to validate the effectiveness of this method.

## 4. Conclusions

In this study, VIS/NIR spectroscopy was used to determine the main chemical compositions in teas and to discriminate the partially fermented teas from different countries and regions. Quantitative analyses of green teas, partially fermented teas, and black teas from Taiwan, Vietnam, and China were performed using VIS/NIR spectra and the SMLR method, and the specific wavelength ranges of constituents that had high correlations with the spectra of tea powders were acquired. The spectral pretreatments removed light scattering, baseline shifts, and other physical interferences on the spectra of tea leaves and powders and enhanced the prediction abilities of the spectral calibration models constructed using SMLR. Satisfactory outcomes were acquired in discriminating the types, origins, and production areas of the Taiwanese and imported teas by applying the SVM method integrated with the specific wavelength ranges of the chemical constituents in tea. The results indicated that NIR could be adopted as an effective method for rapid, nondestructive, and accurate inspection of the constituent concentrations, types, origins, and production areas of tea. This technique could contribute substantially to the quality and safety management of tea.

## Figures and Tables

**Table 1:** The origins of the green, partially fermented, and black tea samples from Taiwan, Vietnam, and China.

Sample Number ^1^	Sample Size ^2^	Types of Tea	Origin	Production Area
G1	3	Green Tea	Taiwan	East
G2	3	Green Tea	Taiwan	North
G3	3	Green Tea	Vietnam	N/A
P1	3	Partially Fermented Tea	Taiwan	South
P2	3	Partially Fermented Tea	Taiwan	South
P3	3	Partially Fermented Tea	Taiwan	South
P4	3	Partially Fermented Tea	Taiwan	North
P5	3	Partially Fermented Tea	Taiwan	East
P6	3	Partially Fermented Tea	Taiwan	South
P7	3	Partially Fermented Tea	Taiwan	South
P8	3	Partially Fermented Tea	Taiwan	East
P9	3	Partially Fermented Tea	Taiwan	South
P10	3	Partially Fermented Tea	Taiwan	North
P11	3	Partially Fermented Tea	Taiwan	North
P12	3	Partially Fermented Tea	Taiwan	East
P13	3	Partially Fermented Tea	Taiwan	South
P14	3	Partially Fermented Tea	Taiwan	South
P15	3	Partially Fermented Tea	Taiwan	North
P16	3	Partially Fermented Tea	Taiwan	North
P17	3	Partially Fermented Tea	China	N/A
P18	3	Partially Fermented Tea	China	N/A
P19	3	Partially Fermented Tea	Vietnam	N/A
P20	3	Partially Fermented Tea	Vietnam	N/A
P21	3	Partially Fermented Tea	Vietnam	N/A
P22	3	Partially Fermented Tea	Vietnam	N/A
P23	3	Partially Fermented Tea	Vietnam	N/A
P24	3	Partially Fermented Tea	Vietnam	N/A
B1	3	Black Tea	Taiwan	East
B2	3	Black Tea	Taiwan	North
B3	3	Black Tea	Taiwan	South
B4	3	Black Tea	Taiwan	South
B5	3	Black Tea	Taiwan	South
B6	3	Black Tea	China	N/A
B7	3	Black Tea	Vietnam	N/A

^1^ G: Green tea; P: Partially fermented tea; B: Black tea. ^2^ The number of the tea samples from the same provenance.

**Table 2:** Concentrations of constituents in green teas, partially fermented teas, and black teas.

Constituents of Tea Leaves ^1^	Type of Tea		
	Green Tea (9) ^2^	Partially Fermented Tea (72) ^2^	Black Tea (21) ^2^
Moisture (%)	5.97 ± 0.60 a ^3^	5.13 ± 1.56 a	6.49 ± 0.81 a
pH	5.62 ± 0.29 a	5.55 ± 0.38 a	4.82 ± 0.17 b
Total Polyphenols (mg/g)	124.63 ± 21.92 a	80.26 ± 14.88 b	68.08 ± 28.70 b
Free Amino Acids (mg/g)	20.22 ± 6.30 a	18.69 ± 8.53 a	16.73 ± 8.97 a
Gallic Acid (mg/g)	3.03 ± 1.90 b	1.84 ± 1.48 b	8.30 ± 3.72 a
GC (mg/g)	68.57 ± 41.12 a	32.05 ± 14.04 b	67.92 ± 27.16 a
EGC (mg/g)	54.31 ± 22.81 a	41.82 ± 27.72 a	2.81 ± 1.53 b
EGCG (mg/g)	51.57 ± 23.28 a	27.81 ± 24.65 b	3.60 ± 5.70 c
ECG (mg/g)	16.78 ± 11.90 a	7.25 ± 4.12 b	5.59 ± 3.94 b
EC (mg/g)	7.68 ± 4.07 a	4.73 ± 2.47 a	2.05 ± 1.86 b
GCG (mg/g)	3.71 ± 1.06 a	3.26 ± 0.88 a	3.14 ± 0.43 a
C (mg/g)	4.98 ± 3.10 a	1.61 ± 0.91 b	0.82 ± 0.61 c
Caffeine (mg/g)	47.39 ± 10.10 a	45.79 ± 15.68 a	55.43 ± 13.06 a

^1^ GC = gallocatechin; EGC = epigallocatechin; EGCG = epigallocatechin gallate; ECG = epicatechin gallate; EC = epicatechin; GCG = gallocatechin gallate; C = catechin. ^2^ Numbers in parentheses represent the number of samples. ^3^ Means within each row followed by the same letter are not significantly different from each other.

**Table 3:** Concentrations of constituents in the partially fermented teas from Vietnam, China, and different production areas in Taiwan (northern, southern, and eastern Taiwan).

Constituents of Tea Leaves	Origin of Partially Fermented Tea					
	Taiwan (48) ^1^				Vietnam (18) ^1^	China (6) ^1^
	All Areas (48) ^1^	North (15) ^1^	South (24) ^1^	East (9) ^1^		
Moisture (%)	5.42 ± 1.27 a ^2^	6.39 ± 1.24 a	4.82 ± 1.02 a	5.43 ± 1.22 a	4.97 ± 2.15 a	3.25 ± 0.54 b
pH	5.63 ± 0.38 a	5.83 ± 0.10 a	5.65 ± 0.38 a	5.23 ± 0.45 a	5.32 ± 0.39 a	5.67 ± 0.02 a
Total Polyphenols (mg/g)	80.10 ± 15.98 a	90.20 ± 12.68 a	80.50 ± 13.66 a	62.18 ± 14.22 b	78.98 ± 15.23 a	85.44 ± 6.27 a
Free Amino Acids (mg/g)	21.46 ± 7.52 a	21.32 ± 7.26 a	22.50 ± 7.73 a	18.93 ± 9.79 b	13.73 ± 9.45 b	11.35 ± 0.56 b
Gallic Acid (mg/g)	1.87 ± 1.59 a	0.91 ± 0.47 a	2.21 ± 1.82 a	2.55 ± 1.86 a	2.12 ± 1.40 a	0.74 ± 0.22 a
GC (mg/g)	33.51 ± 15.74 a	40.44 ± 26.86 a	32.35 ± 6.05 a	25.03 ± 7.32 b	31.86 ± 10.39 a	20.98 ± 0.02 b
EGC (mg/g)	44.53 ± 32.10 a	66.65 ± 42.79 a	37.27 ± 23.94 b	27.01 ± 12.81 b	39.06 ± 18.39 a	28.42 ± 2.30 b
EGCG (mg/g)	26.54 ± 26.17 a	33.61 ± 21.68 a	29.80 ± 30.94 a	6.07 ± 7.01 b	33.83 ± 25.82 a	19.89 ± 1.92 b
ECG (mg/g)	6.68 ± 3.54 b	6.98 ± 3.06 a	7.32 ± 4.27 a	4.45 ± 1.40 b	9.50 ± 5.60 a	5.15 ± 1.29 b
EC (mg/g)	4.77 ± 2.72 a	6.49 ± 3.86 a	4.09 ± 1.80 b	3.72 ± 1.88 b	4.97 ± 2.26 a	3.64 ± 0.76 b
GCG (mg/g)	3.22 ± 0.91 a	3.56 ± 1.51 a	2.93 ± 0.46 a	3.42 ± 0.47 a	3.55 ± 0.94 a	2.76 ± 0.15 a
C (mg/g)	1.58 ± 0.96 a	1.99 ± 1.35 a	1.52 ± 0.79 a	1.03 ± 0.41 a	1.85 ± 0.91 a	1.12 ± 0.25 a
Caffeine (mg/g)	46.14 ± 16.53 a	50.68 ± 29.03 a	43.68 ± 8.84 a	45.16 ± 3.74 a	49.5 ± 14.62 a	31.83 ± 2.89 b

^1^ Numbers in parentheses represent the number of samples. ^2^ Means within each row followed by the same letter are not significantly different from each other.

**Table 4:** Prediction of the constituents’ concentrations in teas using stepwise multiple linear regression models in the wavelength 400–2498 nm.

Constituents of Tea Leaves	Spectrum	Smoothing Points /Derivative Gap	Specific Wavelengths (nm)	1-VR	SECV
Moisture (%)	First Derivative	3/3	2140, 2418, 2326, 932, 2010, 2414	0.98	0.20
pH	First Derivative	3/3	1834, 1310, 750, 460, 1088, 2324	0.97	0.08
Total Polyphenols (mg/g)	First Derivative	3/3	1362, 1676, 1042, 2128, 1036, 2308	0.98	3.42
Free Amino Acids (mg/g)	First Derivative	2/2	2248, 1534, 1828, 1590, 580, 1668	0.88	2.85
Gallic Acid (mg/g)	First Derivative	4/4	564, 632, 1330, 2170, 2346, 1794	0.91	0.99
GC (mg/g)	First Derivative	2/2	1756, 2378, 2358, 2462, 2364, 2406	0.90	7.82
EGC (mg/g)	First Derivative	3/3	596, 2146, 1824, 2346, 1818, 1802	0.87	10.49
EGCG (mg/g)	First Derivative	4/4	2256, 2212, 2136, 1606, 1942, 1662	0.72	13.01
ECG (mg/g)	First Derivative	2/2	664, 2102, 2462, 2122, 2190, 1044	0.83	2.28
EC (mg/g)	First Derivative	2/2	892, 2362, 2360, 2470, 1450, 1304	0.77	1.34
GCG (mg/g)	First Derivative	2/2	2486, 2444	0.08	0.77
C (mg/g)	First Derivative	2/2	2364, 466, 2380, 2204, 1016, 2358	0.87	0.55
Caffeine (mg/g)	First Derivative	6/6	1700, 2332, 1468	0.43	11.11

**Table 5:** Discrimination of the types, origins, and production areas of tea leaves and powders using support vector machine classification analysis.

Class Prediction	Category	Spectrum	Wavelength Range (nm)	Smoothing Points /Derivative Gap	Tea Leaf			Tea Powder		
					Identification Accuracy (%)		Misclassified Sample	Identification Accuracy (%)		Misclassified Sample
					Calibration Set	Prediction Set		Calibration Set	Prediction Set	
Case 1	Green Tea	First Derivative	450–900 1000–1400 1600–1850 2100–2400	3/3	100	100	None	100	100	None
	Partially Fermented Tea									
	Black Tea									
Case 2	Taiwanese Tea	First Derivative	1500–1900 2200–2300	2/2	100	100	None	100	100	None
	Imported Tea									
Case 3	Taiwanese Tea	First Derivative	400–2498	2/2	100	86	P24	100	86	P24
	Vietnamese Tea	First Derivative	1500–1900 2200–2300	2/2	100	100	None	100	100	None
Case 4	Taiwanese Tea	First Derivative	400–2498	2/2	100	83	P17	100	83	P17
	China Tea	First Derivative	450–900 1600–1850 2100–2400	2/2	100	100	None	100	100	None
Case 5	Northern Taiwanese Tea	First Derivative	400–2498	3/3	100	83	P14	100	83	P14
	Southern Taiwanese Tea	First Derivative	450–900 1000–1400 1600–1850 2100–2400	3/3	100	83	P14	100	83	P14
	Eastern Taiwanese Tea	First Derivative	1500–1900 2200–2300	3/3	100	67	P5, P14	100	67	P5, P14
		First Derivative	450–900 1000–1400 1500–1900 2100–2400	3/3	100 ^1^	100 ^1^	None ^1^	100 ^1^	100 ^1^	None ^1^

^1^ The P5 sample was reassigned as a southern Taiwanese tea, and the sample of P14 was reassigned as an eastern Taiwanese tea, after which the SVM analyses of the tea leaves and powders were performed again.
